# Reduced quality of life, more technical challenges, and less study motivation among paramedic students after one year of the COVID-19 pandemic – a survey study

**DOI:** 10.1186/s12909-023-04120-8

**Published:** 2023-03-01

**Authors:** Kristin Häikiö, Astrid Karina Harring, Rune Kveen, Kim Rand, Trine Møgster Jørgensen

**Affiliations:** 1 Faculty of Health Sciences, Department for Prehospital Work, Oslo Metropolitan University – OsloMet, Oslo, Norway; 2 Division of Prehospital Services, Oslo University Hospital (OUH), Oslo, Norway; 3 HØKH – Health Services Research Unit, Akershus University Hospital, Lørenskog, Norway

**Keywords:** Pandemic, COVID-19, Emergency medical technicians, Paramedic, Quality of life, Allied Health Personnel, student, online education, motivation

## Abstract

**Introduction:**

Despite the lack of knowledge about the SARS-CoV2 virus, the lack of personal protection gear among frontline healthcare workers, and lack of vaccines in the beginning of the pandemic, paramedic students in Norway contributed to the National response against the COVID-19 pandemic by working in test-stations, ambulance services, ambulance decontamination stations etc. Despite fear of contracting the COVID-19 reported by healthcare workers worldwide, paramedic students in Norway reported higher-than-average quality of life after four months of the COVID-19 pandemic (first pandemic wave). In this study we aimed to investigate how students reported their quality of life, study motivation and job satisfaction after one year of living with the COVID-19 pandemic.

**Method:**

At two data collection point, all paramedic students enrolled at Oslo Metropolitan University were invited to participate in a digital, online, self-administered survey. The first data collection was in June 2020 (the first pandemic wave), while the second data collection was in March 2021 (the third pandemic wave). Results from both samples were analyzed independently with descriptive statistics. Differences between the groups were analyzed using an independent T-test and Mann–Whitney-U test to discover changes over time. Multiple linear regression analysis was used to estimate the difference attributable to timing (first vs. the third wave), seniority, and student gender.

**Results:**

The samples consist of slightly more female students than male students. The mean age in both samples is 24.6 years. Despite the higher-than-average level of quality of life in the first pandemic wave, results show that there was a significant reduction in students’ health-related quality of life (*p* < 0.001, B -0.059, SE 0.016), study motivation (*p* = 0.002, 95% CI:0.09,0.41), and job satisfaction (*p* = 0.005, 95% CI:1.62,9.00) after the third pandemic wave in Norway. Surprisingly, students experienced more technical challenges in the third wave, e.g., poor internet connection, sound pollution, and poor picture quality, despite more experience among students and teachers.

**Conclusion:**

Our results show that paramedic students had significant worsening experiences in the late pandemic wave compared to the first pandemic wave. Universities and governments should learn from the COVID-19 pandemic to develop better preparedness plans for future pandemics and knowledge about students' well-being should be considered in future preparedness plans for higher education and the government plans for the education of front-line healthcare workers during a pandemic to facilitate the continuation of necessary education.

## Introduction

Global pandemics have been expected and are expected in the future [1]. In Norway, as in many other countries, universities closed during the COVID-19 pandemic [2]. Social distancing was required, travel restrictions were applied, and students suddenly attended online, digital teaching [3]. As a result, students around the world have reported high levels of anxiety and stress [3–6], and a Norwegian study reported that one in three students felt lonely, one in four were unhappy with their lives, and many struggled with psychological problems such as stress and anxiety [7–9].

Although there are many studies of students’ responses to COVID-19 [10], few have been conducted on paramedic students [10, 11] and even fewer have followed students experiences one year into the pandemic. In a previous paper, it is reported that despite a lack of personal protection equipment in the early pandemic phase, Norwegian paramedic students participated in the national COVID-19 response both through clinical and administrative work [10]. Surprisingly, the students reported higher levels of health-related quality of life (HRQoL) than the general Norwegian population in the same age groups [10]. Although some recent studies have described resilience among paramedics during the COVID-19 pandemic [11], increased levels of stress, anxiety, and emotional labour for paramedics during the pandemic have also been reported [12, 13].

This paper investigates changes in paramedic students' self-reported HRQoL, study motivation, technical challenges, and job satisfaction between June 2020 (first wave) and March 2021 (third wave) of the SARS-CoV-2 pandemic in Norway. Results can be utilized to understand students' experiences better and enable universities and governments to prepare for future pandemics and develop local and national preparedness plans.

## Method

### Study design

**Fig. 1:**
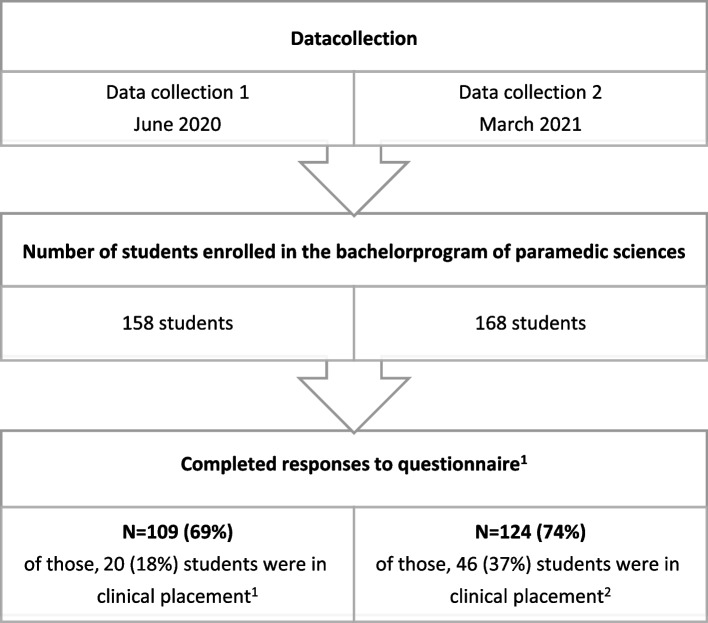
Overview of the data collection and student participation. ^1^Questionnaire for all students: self-reported health-related quality of life (HRQoL), study motivation and technical challenges; ^2^in addition to the first questionnaire, students in clinical placement reported their job satisfaction

This study reports on two retrospective cross-sectional data collections by self-administered, online, surveys of paramedic students in Norway. The first data collection (DC1) was conducted in June 2020, details of which can be found elsewhere [2, 10]. The second data collection (DC2) was conducted in March 2021. Aside from the timing, the survey and recruitment process in DC1 and DC2 were identical. At both data collection points, all students enrolled in the bachelor’s program in paramedic science at Oslo Metropolitan University (OsloMet) were invited to participate (Fig. 1), meaning students’ seniority had increased by a year in DC2. Consequently, the third-year students from DC1 were not part of DC2, and the first-year students in DC2 were not part of DC1. The variables of interest for this paper differ from those reported earlier, except for HRQoL. HRQoL was reported from DC1 in a previous paper [10].

### The context

158 and 168 students were enrolled in the bachelor’s program in paramedic science at OsloMet at the time of DC1 and DC2, respectively, meaning the response rates were 69% (DC1) and 74% (DC2). The bachelor’s program offers theoretical subjects with additional simulation training, and one-third of the program is dedicated to supervised clinical placements. The clinical placements were mainly undertaken in the pandemic's Norwegian epicentre, in the Oslo University Hospital ambulance department (OUH). Further information about the context is available in our previous paper [10].

Norway's emergency medical system (EMS) is a government-funded non-physician-based system. The education of ambulance personnel is either a three-year bachelor’s programme (180 ECTS) followed by a paramedic authorization [14] or a two-year high school education along with two years of EMS practice before gaining their authorization as an Emergency Medical Technician (EMT) [15]. The EMT authorization can also be obtained for nurses after working two years in the EMS and completing compulsory advanced life support courses [15]. Post authorization EMTs can enrol to further education programs.

### Data collection

For DC2, similar to DC1, all students enrolled in the bachelor’s programme in paramedic science in March 2021 were invited to an electronic survey by email. Three reminders were sent.

Variables of interest were chosen before DC1 [10], based on previous research on the effect of the COVID-19 on healthcare workers, such as level of anxiety, depression, and distress [8].

### Data variables

#### Student characteristics

We collected information on students’ gender, age, and seniority (study year).

#### Health-related quality of life

Health-related quality of life (HRQoL) was measured using Euroqol’s EQ-5D-5L instrument in Norwegian translation [16]. The instrument is widely tested and validated [17] and translated to Norwegian among other languages [18, 19]. The instrument has two parts. The first is the descriptive part which results in a number between 0–1 (0 = death, 1 = full health). We refer to this number as the EQvalue. The second is a *visual analog scale* between 0–100 (0 = worst possible health, 100 = best possible health) and is referred to as EQvas [20].

#### Students’ change in study motivation

Students were asked whether their study motivation had changed because of the pandemic. Their answers included changes in the negative direction (-1), no change (0), or changes in a positive direction (1). The variable is interpreted as linear from -1 to 1.

#### Technical challenges

The survey included questions regarding whether students experienced technical/digital challenges during the digital lectures (0 = no, 1 = yes): difficulties concerning assessing digital platforms, poor internet connection, digital sound pollution, poor digital picture, lack of headset/speaker, lack of camera, lack of knowledge regarding how to connect (e.g. to digital platforms/meetings), lack of necessary software, lack of available computer, compatible phone or tablet, and difficulties reading on their cell phone.

#### Students’ job satisfaction in clinical placement

For students in clinical placement, we included the Job Satisfaction Scale (JSS) [21], which contains ten questions on a Likert scale ranging from 1–7, where 1 equals extremely dissatisfied and 7 equals extremely satisfied. A few students in clinical placement were paid for their work due to an extraordinary pandemic regulation [10].

### Statistical analysis

R for windows version 4.0.4 was used for linear regression modelling of HRQoL, wheras other statistical analyses were performed in SPSS (Statistical Package for the Social Sciences), version 26. Descriptive statistics describing participants’ characteristics, HRQoL, changes in study motivation, and job satisfaction were performed. Mean and median values (including standard deviations) are presented for normally and skewed distributed variables. Minimum- and maximum values for students’ age are not reported due to the risk of identifying participants.

Independent samples t-tests with 95% confidence intervals were used to investigate differences between groups. When the assumptions of normality or homogeneity were violated, and when the number of cases was deficient, we used the Mann–Whitney U test as sensitivity analysis. In addition, Levene's Test for Equality of variances was used to investigate the homogeneity of the groups. For HRQoL we also investigate changes in the cohorts, meaning we compared the results from first- and second year students in DC1 to second- and third-year students in DC2.

Because most students were in ordinary, unpaid, clinical placement, there were many missing values in the JSS items which regarded students' satisfaction with salary. Therefore, to calculate the total score for the scale with missing values on one item, we applied predicted imputation based on the mean scores of the sample for the corresponding item [22].

### Ethics

Participation was voluntary, and the Regional Committees for Medical and Health Research Ethics South-East D approved a second data collection for this study (DC2) (reference no. 142135).

The Norwegian Centre approved the data management plan for Research Data (reference no. 409603). All data was encrypted and directly loaded to Tjenester for Sensitive Data (TSD) for storage and analysis [23].

## Results

### Characteristics for the sample

The characteristics of the samples are described in Table 1.

**Table 1:** Characteristics of the samples

	Data collection 1, *N* = 109		Data collection 2, *N* = 124	
Female gender, n (%)	62	(56.9)	72	(58.1)
Male gender, n (%)	47	(43.1)	52	(41.9)
Age, mean (sd)	24.6	(4.2)	24.6	(4.6)
Student seniority – 1^st^ year, n (%)	42	(38.5)	39	(31.5)
Student seniority – 2^nd^ year, n (%)	44	(40.4)	39	(31.5)
Student seniority – 3^rd^-year n, (%)	23	(21.1)	46	(37.1)

### Health-related quality of life

The mean values of HRQoL (EQvalue and EQvas) were lower in DC2 (Table 2).

**Table 2:** Health-related quality of life (EQvalue and EQvas) for both data collection

	Data collection 1		Data collection 2	
EQvalue, mean (sd)	0.92	(0.12)	0.76	(0.15)
EQvas mean (sd)	82.9	(12.0)	87.0	(13.0)

Multiple regression analysis showed that after adjusting for the effect of student seniority (Table 3), EQvalue and EQvas were significantly lower in DC2 (EQvalue: *p* < 0.001, B -0.059, SE 0.016) (EQvas: *p* < 0.001, B -7.554, SE 1.797) compared to DC1.

**Table 3:** Results of multiple linear regression analysis of HRQoL

	**EQVALUE**				**EQVAS**			
Predictor	Coef	(SE)	*p*	sign	Coef	(SE)	*p*	sign
3rd year (base case)	0.927	(0.019)	< 0.001	*	83.349	(2.163)	< 0.001	*
2nd year	-0.013	(0.020)	0.528		0.200	(2.230)	0.928	
1st year	-0.040	(0.020)	0.047	*	-2.819	(2.239)	0.209	
Data collection 2	-0.059	(0.016)	< 0.001	*	-7.554	(1.797)	< 0.001	*
Male gender	0.033	(0.016)	0.041	*	1.304	(1.789)	0.467	

^*^ Statistically significant value (< 0.05)

**Fig. 2:**
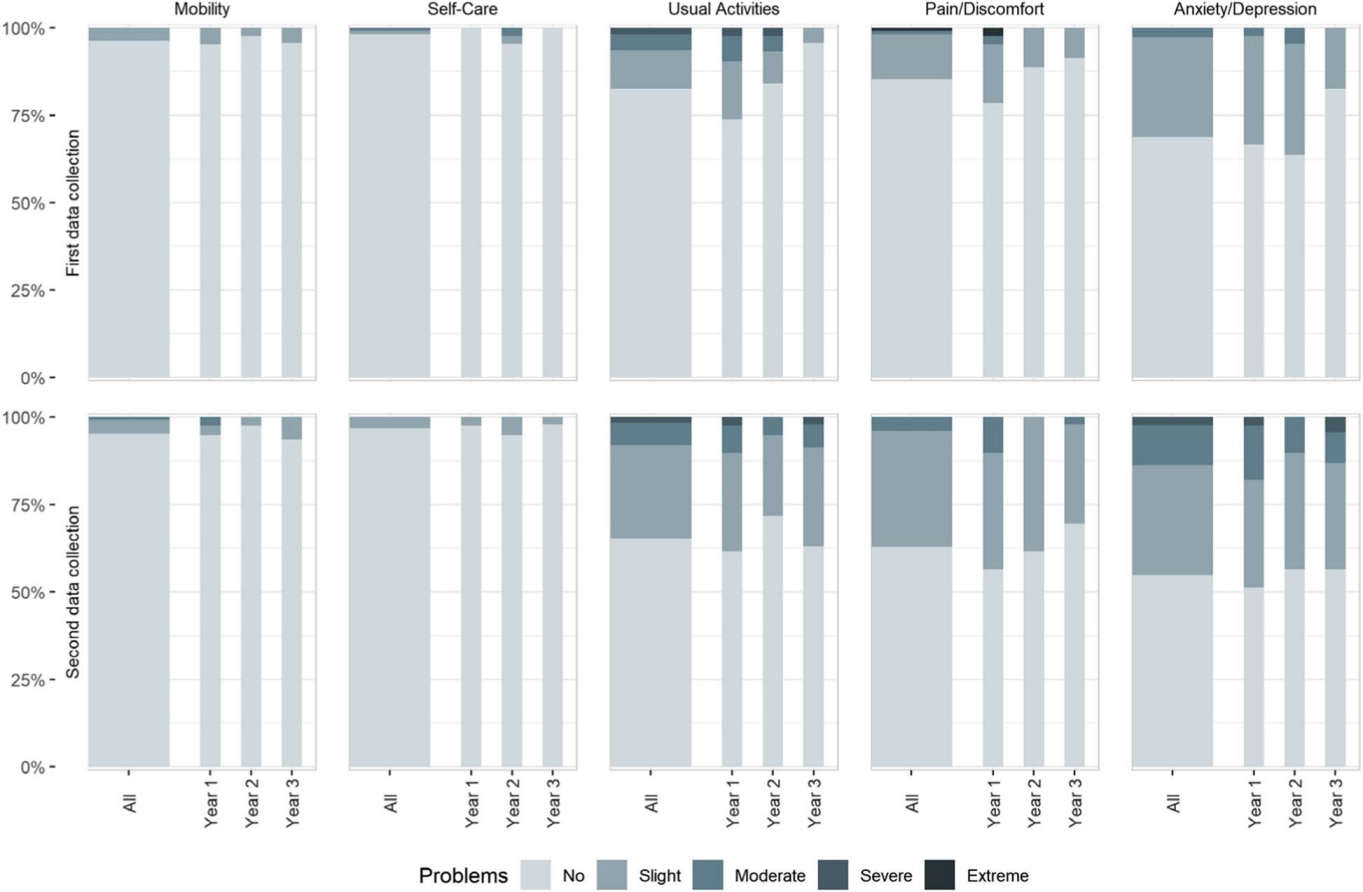
Paramedic students’ HRQoL scores on each of the five domains, stratified by study year

The mean scores were lower for all cohorts (classes) in DC2 when we followed each cohort (class) from 2020 to 2021. Our DC2 students reported more problems in all dimensions. The difference was most evident in the two domains pain/discomfort and anxiety/depression (Fig. 2).

### Changes in students’ study motivation

In the first data collection (DC1), 42.1% (*n* = 47) of the students reported a negative change in study motivation because of the COVID-19 pandemic, and 11.0% (*n* = 12 of 109) reported a positive change in study motivation. In the DC2, 62.1% (*n* = 77) of the students reported a negative change in study motivation, while only 4.8% (*n* = 6 of 124) reported a positive change. There was a statistically significant drop in students’ study motivation due to the pandemic in March 2021 compared to June 2020 (*p* = 0.002, 95% CI:0.09,0.41).

### Technical challenges related to digital teaching during the COVID-19 pandemic

The four most commonly experienced technical difficulties were poor internet connection, sound pollution, poor picture, and accessing platforms. Independent T-tests and Mann–Whitney-U tests confirmed that the prevalence of experienced technical difficulty regarding digital teaching was significantly worse in the DC2 (March 2021) compared to DC1 (June 2020) in three out of four areas, see Table 4.

**Table 4:** Students reported technical challenges related to digital teaching for the four most common challenges

	Data collection 1		Data collection 2		Change	95% Confidence interval		*P*-value
						low	high	
Poor internet connection, n (%) *	19	(17.4)	42	(33.9)	↑	-0.277	-0.052	0.004
Sound pollution, n (%) *	14	(12.8)	41	(33.1)	↑	-0.307	-0.097	0.000
Poor picture quality, n (%) *	5	(4.6)	28	(22.6)	↑	-0.264	-0.096	< 0.001
Difficulty accessing digital platforms, n (%) **	12	(11)	12	(9.7)	↓			0.74

^*^p-values are based on t-tests, and significant differences were confirmed by Mann Whitney U, resulting in equal *p*-values

^**^ *p*-value is based on the parametric test Mann Whitney-U

### Job satisfaction

Students in clinical placement reported their job satisfaction. In DC1, 20 students (18.3%) were in clinical placement and reported a mean JSS score of 58.5 (sd 5.4), whereas the number of students for the DC2 was 46 (37.1%). The independent sample T-test showed that for the combined JSS score, the mean value was statistically significantly lower in DC2 (*p* = 0.005, 95% CI:1.62, 9.00). The JSS sum score was normally distributed, but Levene’s Test for Equality of Variances showed that homogeneity was violated (*p* = 0.040). Sensitivity analysis with the Mann–Whitney U test confirmed the significant difference between groups (*p* = 0.027).

As seen in Table 5, all but two parameters in the JSS were reduced in the DC2.

**Table 5:** Results from Job Satisfaction Scale

	Data collection 1 *n* = 20		Data collection 2 *n* = 46	
	mean	(sd)	mean	(sd)
JSS1 amount of responsibility you are given	5.9	(1.1)	5.8	(1.2)
JSS2 variation on work	6.0	(1.0)	5.7	(1.3)
JSS3 your colleagues and fellow workers	6.8	(0.4)	5.8	(1.4)
JSS4 your physical working conditions	6.3	(0.9)	5.6	(1.3)
JSS5 your opportunities to use your skills	6.1	(0.9)	5.5	(1.4)
JSS6 your overall job situation	6.5	(0.8)	5.7	(1.2)
JSS7 freedom to choose your own methods of working	5.3	(1.3)	4.9	(1.6)
JSS8 the recognition you get for good achievements	6.0	(1.2)	5.2	(1.9)
JSS9 your rate of pay	3.5	(0.8)	3.8	(1.0)
JSS10 your work hours	6.2	(0.5)	5.2	(1.4)
Mean JSS sum score	58.5	(5.4)	53.2	(9.3)

## Discussion

### Health-related quality of life dropped between the first and third pandemic wave

Regarding the level of HRQoL in DC1 we have earlier reported that the average level of HRQoL was higher for paramedic students compared to the general population of the same age [10]. However, there was a significant drop in students' HRQoL levels in DC2, which aligns with previous research findings from front-line healthcare providers, e.g. in China [24] and Serbia [9], nursing students in the US [25], and paramedics in Wales [13]. A study among nursing students in Norway reported that their quality of life was lower during the COVID-19 pandemic than in the pre-pandemic period [26]. A study from the United Kingdom (UK) in June/July 2020 found that for bachelor undergraduates the mean EQvalue was 7.88 (sd 2.65). This is similar to our findings in the DC2 (EQvalue 0.76 (sd 0.15)), but was measured at the same time as our DC1 and in a different country with different healthsystems.

A study from Poland investigating stress and resilience among paramedics found that paramedics experienced lower subjective stress during the COVID-19 pandemic than before the pandemic. This lower subjective stress is surprising due to the mental stress reported by healthcare workers elsewhere. A systematic review examining resilience among healthcare workers during the COVID-19 pandemic found that coping behaviours, resilience and social support were associated with positive psychological and mental health outcomes [27]. Therefore, a potential explanation suggested by the authors was that paramedics might have sufficient psychological resilience resources to handle challenging situations and settings [11]. In addition to good health among paramedic students, the activation of resilience may have contributed to a high level of HRQoL at the beginning of the pandemic (DC1). However, the activation is exhausting in the long run [11] and may explain the decrease in HRQoL in the DC2. This explanation aligns well the Yerkes-Dodson law stating that there is an optimal level of stress corresponding to an optimal level of performance [28]. The association between stress and performance is presented as an inverted U-shaped curve where low stress is associated with low performance quality, and entails boredom or apathy; optimal level of stress entails an optimal level of performance quality; and too high level of stress is associated with low performance quality and entails high anxiety [28]. During the COVID-19 pandemic students had to deal with many sudden changes which may have exceeded their optimal stress levels and consequently reduced their HRQoL. Examples of such stressors are online education instead of classroom education, closed universities and social distancing.

The level of HRQoL in DC1 was higher among paramedic students five months into the pandemic compared to the general Norwegian population prior to the pandemic [10]. Resilience among paramedic students might explain it, but various other factors may also play a role in these high HRQoL scores, which contradict other research findings of healthcare workers and healthcare students during the pandemic [13]. First is the Scandinavian welfare model, which offers a "safety net" for the population in case of sickness and loss of income. Second, is the successful handling of the pandemic in Norway, and third, is the low pandemic-related mortality in Norway. Also, there may be a selection of particularly healthy students for paramedic education, effectively masking some decrease in HRQoL scores (no baseline data on paramedic students were available) [10].

At the time of our DC2, students had dealt with online teaching and social distancing for one year. Consequently, many students in Norway reported loneliness, anxiety, and reduced quality of life [7]. On the other hand, knowledge about the Corona-virus, the way it spreads, and the risk factors for becoming seriously sick improved considerably between DC1 and DC2. Vaccination had also begun, though predominantly for the elderly, and the supply of personal protection gear was no longer insufficient. It is reasonable to assume that these factors could have reduced anxiety about the virus and the disease. However, the reported reduction in HRQoL between DC1 and DC2 suggests that anxiety reduction appears to have been offset by other factors.

Study motivation, technical challenges and HRQoL during Covid-crises – different sides of the same phenomenon?

Although previous research has shown that online teaching may support student learning and satisfaction with studies [29], our students’ motivation dropped significantly after the closing of the university and the change from classroom teaching to online platforms. Australian paramedic students also reported difficulties adjusting to online learning and getting into a routine [2]. Consequently, it is no surprise that student motivation decreased during the COVID-19 pandemic with long-time social distancing and sudden changes from classroom teaching to online platforms.

The main benefits of online teaching are flexibility and time efficiency. However, a study of medical students in the United Kingdom (UK) found that students do not perceive it as engaging, enjoyable, or effective as face-to-face learning [30]. The home environment and lack of designated study space became barriers to online education, and family distraction and poor internet connection were the most common issues [30]. Surprisingly, more of our students experienced technical problems in the DC2 compared to DC1. As teachers and students gained experience with digital platforms, we expected fewer technical problems and challenges. However, students might also have expected teachers to advance their skills and, therefore, may have had reduced patience with technical difficulties and developed higher demands over time. It could also be that the staff did not adapt or variate teaching methods enough. Dost and Hossain [30] found that by using team-based or problem-based learning in digital groups, students were more satisfied and motivated and had more significant learning outcomes.

Other factors may also have contributed to the measured decline in study motivation. For instance, studies prior to the COVID 19 pandemic have shown positive correlations between HRQL dimensions and study motivation among medical- and nursing students [31, 32]. A disproportional balance between demands, available resources, and students' ability to cope could lead to mental exhaustion and fatigue, which has been shown to negatively affect study motivation, mental health, and academic performance [33, 34]. Further investigations in factors that could interact with study motivation could inform areas such as selection of students to paramedic education, application of teaching methods, promoting resilience, self-care and strategies for coping during/with hardship.

### Job satisfaction declined from the first to the third pandemic wave

Many domains that showed a negative change regarding job satisfaction were related to human factors. For example, reduced satisfaction with colleagues and lack of recognition for achievements suggest that the ambulance personnel guiding the students in their clinical placements were fatigued and not as attentive and supportive as they usually are. After all, the UK study found that 2/3 of paramedics suffered from burnout [35].

Results from the JSS indicated that physical working conditions and work hours worsened between DC1 and DC2. A study of paramedics in the UK reported that paramedics felt a widespread disruption at work and that inconsistent information and rapidly changing procedures, as well as the increased demand for ambulances and the comprehensive disinfection afterwards, led to exhaustion [35]. Thus, organizational and administrative factors that could ease the burden not only for our students, but the EMS at large, need to be addressed.

### Strengths and limitations

This study is one of few studies investigating paramedic students and comparing students' experiences with the COVID-19 pandemic between different pandemic waves. The survey is based on validated instruments and scales. OsloMet is the largest paramedic education in Norway and a leading paramedic education internationally, and we have a high participation rate in both data collections. However, the students from OsloMet are not representative of Norway or elsewhere. The participants lived and studied in the epicentre of Norway and at a location where hospitals are short driving distances from where patients live. For ambulances in other locations, the geography is different. Consequently, the results cannot be generalized. Despite the lack of generalisability, the results provide valuable insights that we believe have value outside our study population.

## Conclusion

Our results align with international literature indicating that healthcare workers and healthcare students have experienced negative changes in quality of life, satisfaction, and motivation during the COVID-19 pandemic. Our study adds to the existing research field that paramedic students had a high level of health-related quality at the beginning of the pandemic and a significant worsening experience in the later pandemic wave. Students' well-being should be considered in future preparedness plans for higher education with respect to educate front-line healthcare workers during a pandemic. The continuation of education of front-line healthcare workers is important to meet future demands, and even more so during a health-crisis.

## Data Availability

The datasets generated or analyzed during the current study are not publicly available due to the risk of identification of individuals when variables are combined. However, a limited dataset with one or a few variables may be available from the corresponding author on request.

## Abbreviations

COVID-19: Coronavirus disease of 2019
DC1: First data collection (June 2020)
DC2: Second data collection (March 2021)
HRQoL: Health-related quality of life
JSS: Job satisfaction scale
OsloMet: Oslo Metropolitan University
OUH: Oslo University Hospital
UK: United Kingdom

## References

[1] World Health Organization. Preparing for pandemics [online]: WHO; 2022 [cited 2023 feb 28]. Available from: https://www.who.int/westernpacific/activities/preparing-for-pandemics.

[2] Meeter M, Bele T, Hartogh C d, Bakker TC, de Vries RE, Plak S. College students’ motivation and study results after COVID-19 stay-at-home orders [Internet]. PsyArXiv; 2020. Available from: psyarxiv.com/kn6v9.

[3] Sahu P (2020). Closure of universities due to Coronavirus disease 2019 (COVID-19): impact on education and mental health of students and academic staff. Cureus.

[4] Al-Rabiaah A, Temsah MH, Al-Eyadhy AA, Hasan GM, Al-Zamil F, Al-Subaie S (2020). Middle East Respiratory Syndrome-Corona Virus (MERS-CoV) associated stress among medical students at a university teaching hospital in Saudi Arabia. J Infect Public Health.

[5] Batais MA, Temsah MH, AlGhofili H, AlRuwayshid N, Alsohime F, Almigbal TH (2021). The coronavirus disease of 2019 pandemic-associated stress among medical students in middle east respiratory syndrome-CoV endemic area: an observational study. Medicine (Baltimore).

[6] Hawley SR, Thrivikraman JK, Noveck N, St.Romain T, Ludy M-J, Barnhart L (2021). Concerns of college students during the COVID-19 pandemic: thematic perspectives from the United States, Asia, and Europe. J Appl Learn Teach.

[7] Folkehelseinstituttet (FHI) (The public health institute). Livskvalitet og psykisk helse under koronapandemien november - desember 2020 [online]: Folkehelseinstituttet; 2020 [updated Dec 17 2020; cited 2023 Feb 28]. Available from: https://www.fhi.no/div/helseundersokelser/fylkeshelseundersokelser/livskvalitet-og-psykisk-helse-under-koronaepidemien--nov-des-2020/.

[8] Lai J, Ma S, Wang Y, Cai Z, Hu J, Wei N (2020). Factors associated with mental health outcomes among health care workers exposed to Coronavirus disease 2019. JAMA Netw Open.

[9] Stojanov J, Malobabic M, Stanojevic G, Stevic M, Milosevic V, Stojanov A (2021). Quality of sleep and health-related quality of life among health care professionals treating patients with Coronavirus disease-19. Int J Soc Psychiatr.

[10] Häikiö K, Andersen JV, Bakkerud M, Christiansen CR, Rand K, Staff T. A retrospective survey study of paramedic students’ exposure to SARS-CoV-2, participation in the COVID-19 pandemic response, and health-related quality of life. Scand J Trauma Resusc Emerg Med. 2021;29(1):153. 10.1186/s13049-021-00967-2 PMC8522862 34663422

[11] Piotrowski A, Makarowski R, Predoiu R, Predoiu A, Boe O (2021). Resilience and subjectively experienced stress among paramedics prior to and during the COVID-19 pandemic. Front Psychol.

[12] Hayes C, Corrie I, Graham Y (2020). Paramedic emotional labour during COVID-19. J Paramed Pract.

[13] Rees N, Smythe L, Hogan C, Williams J (2021). Paramedic experiences of providing care in Wales (UK) during the 2020 COVID-19 pandemic (PECC-19): a qualitative study using evolved grounded theory. BMJ Open.

[14] Forskrift om nasjonal retningslinje for paramedisinutdanning (paramedisinforskriften), FOR-2020–01–31–99. 2020 [cited 2023 feb 28]. Available from: https://lovdata.no/dokument/SF/forskrift/2020-01-31-99.

[15] The Norwegian directorate of health. Ambulansearbeider - Norge The Norwegian directorate of health [online]: Norwegian directorate of health; 2019 [updated 2022 nov 22; cited 2023 feb 28]. Available from: https://www.helsedirektoratet.no/tema/autorisasjon-og-spesialistutdanning/autorisasjon-og-lisens?path=1-1-ambulansearbeider-norge.

[16] EuroQol Group. EQ-5D-5L Self-complete version on Digital Devices [online]: EuroQol; 2021 [cited 2023 feb 28]. Available from: https://euroqol.org/eq-5d-instruments/eq-5d-5l-available-modes-of-administration/self-complete-on-digital/?_gl=1*ziuyi5*_up*MQ..*_ga*MzI3Njc2ODY3LjE2Nzc1NzM3NDU.*_ga_02T9YV6MT2*MTY3NzU3Mzc0NS4xLjEuMTY3NzU3MzgwMS4wLjAuMA.

[17] Feng Y-S, Kohlmann T, Janssen MF, Buchholz I (2021). Psychometric properties of the EQ-5D-5L: a systematic review of the literature. Qual of Life Res.

[18] Garratt AM, Hansen TM, Augestad LA, Rand K, Stavem K (2022). Norwegian population norms for the EQ-5D-5L: results from a general population survey. Qual Life Res Int J Qual Life Aspects Treat, Care Rehabil.

[19] Stavem K, Augestad L, Kristiansen I, Rand K (2018). General population norms for the EQ-5D-3L in Norway: comparison of postal and web surveys. Health Qual Life Outcomes.

[20] EuroQol Group. EQ-5D Instruments, Abaout EQ-5D [online]: EuroQol Group; 2017 [cited 2018 05.09.18]. Available from: https://euroqol.org/eq-5d-instruments/eq-5d-5l-about/.

[21] Warr PCJ, Wall T (1979). Scales for the measurement of some work attitudes and aspects of psychological well-being. J Occup Psychol.

[22] Çokluk Ö, Kayri M (2011). The effects of methods of imputation for missing values on the validity and reliability of scales. Educ Sci Theory Pract.

[23] University of Oslo. About TSD [online]: University of Oslo; [cited 2023 feb 28]. Available from: https://www.uio.no/english/services/it/research/sensitive-data/about/index.html.

[24] Lai J, Ma S, Wang Y, Cai Z, Hu J, Wei N (2020). Factors associated with mental health outcomes among health care workers exposed to Coronavirus disease 2019. JAMA Network Open.

[25] Keener TA, Hall K, Wang K, Hulsey T, Piamjariyakul U (2021). Quality of life, resilience, and related factors of nursing students during the COVID-19 pandemic. Nurse Educ.

[26] Beisland EG, Gjeilo KH, Andersen JR, Bratås O, Bø B, Haraldstad K (2021). Quality of life and fear of COVID-19 in 2600 baccalaureate nursing students at five universities: a cross-sectional study. Health Qual Life Outcomes.

[27] Labrague LJ (2021). Psychological resilience, coping behaviours and social support among health care workers during the COVID-19 pandemic: a systematic review of quantitative studies. J Nurs Manag.

[28] Nickerson C. The yerkes-dodson law and performance [online]: SimplyPsychology; 2021 [Updated 2015 nov 15; cited 2023 feb 28]. Retrieved from: https://www.simplypsychology.org/what-is-the-yerkes-dodson-law.html.

[29] Baber H. Determinants of Students’ Perceived Learning Outcome and Satisfaction in Online Learning during the Pandemic of COVID19. J Educ e-Learn Res. 2020;7(3):284–92

[30] Dost S, Hossain A, Shehab M, Abdelwahed A, Al-Nusair L (2020). Perceptions of medical students towards online teaching during the COVID-19 pandemic: a national cross-sectional survey of 2721 UK medical students. BMJ Open.

[31] Henning MA, Krägeloh CU, Hawken SJ, Doherty I, Zhao Y, Shulruf B (2011). Motivation to learn, quality of life and estimated academic achievement: medical students studying in New Zealand. Med Sci Educ.

[32] Elbasuony M (2016). Correlation between academic motivation to study nursing and health-related quality of life among nursing students. J Am Sci.

[33] Schaufeli WB, Bakker AB (2004). Job demands, job resources, and their relationship with burnout and engagement: a multi-sample study. J Organ Behav.

[34] van Zyl LE, Rothmann S, Zondervan-Zwijnenburg MAJ (2021). Longitudinal trajectories of study characteristics and mental health before and during the COVID-19 lockdown. Front Psychol.

[35] Petrie K, Smallwood N, Pascoe A, Willis K (2022). Mental health symptoms and workplace challenges among Australian paramedics during the COVID-19 Pandemic. Int J Environ Res Public Health.

